# Beyond gratitude: a qualitative investigation of compliment letters received by a neonatology service

**DOI:** 10.1186/s12913-026-14041-z

**Published:** 2026-01-24

**Authors:** Valérie Clerc, Friedrich Stiefel, Béatrice Schaad, Céline Bourquin

**Affiliations:** 1 Psychiatric Liaison Service, Lausanne University Hospital (CHUV), University of Lausanne, Les Allières, Av. de Beaumont 23, Lausanne-CHUV, 1011 Switzerland; 2 Center for Patient, Family and Professional Experiences, Lausanne University Hospital (CHUV) and University of Lausanne, Rue du Bugnon 21, Lausanne-CHUV, Lausanne, 1011 Switzerland

**Keywords:** Compliment letters, Patients, Satisfaction, Gratitude, Neonatology

## Abstract

**Background:**

Patients’ and relatives’ feedback is used by hospitals to improve the quality and safety of care. It can be solicited, as in the case of satisfaction questionnaires or complaints collected by mediation centers. Hospital users also provide unsolicited feedback by writing compliment letters, which are generally considered as expressions of gratitude. This study investigated compliment letters received by a neonatology service from parents with a twofold objective: first, to investigate compliment letters’ content regarding parents’ experiences and second, to explore underlying needs that motivated parents to provide feedback.

**Methods:**

This qualitative descriptive study was based on the analysis of 573 compliment letters collected by the neonatology service of Lausanne University Hospital in Switzerland between 2009 and 2020. The parents’ lived experiences contained in compliment letters were analyzed using framework analysis. The needs and functions that their feedback fulfilled were examined through a custom analysis method, combining psychological and socio-anthropological perspectives, and informed by the results of the framework analysis.

**Results:**

Five core themes characterized parents’ experiences: recall of the care and hospital stay (contextual information to refer to the children’s stays in the service), life after the hospital discharge (children’s health evolution and development), parents’ feelings (how parents experienced their children’s care), perception of the healthcare professionals (healthcare professionals ways of being and doing), parents’ gratitude (thanks to healthcare professionals for their acts or their qualities). Five underlying needs were identified: thanking the team, closure (claim that the children were no longer patients), partial closure (feedback on the state of the children), taking its own place in the care (parent’s account of the impact of children’s hospitalization), managing the sense of indebtedness (parents’ acknowledgement that they owe something to the team).

**Conclusion:**

When parents write compliment letters, they mostly express how they experienced their children’s hospitalization and how they managed to make sense out of it. Compliment letters thus have other functions than expressing gratitude. They allow parents to sustain relationships with healthcare professionals, support the normalization of their children’s situation and their recovery or provide testimony of experiences. They contain important information about the writers, health care professionals, the relationship they have established, hospital operations and healthcare organization. As hospital users' experiences are thought to contribute to health care quality, it matters that health services consider the information they convey.

## Background

Patients and relatives’ feedback after a hospital stay provides useful information to improve health services [1]. Hospitals solicit this feedback by means of satisfaction questionnaires [2–5] or complaints collected by mediation centers [6, 7]. However, hospital users might also provide unsolicited feedback in the form of gifts, cards, letters, emails and accounts of experiences on social media.

Negative feedback, as in the case of complaints, is currently particularly valued in health care institutions because it allows access to the unique view of patients and their relatives [8–12] and therefore contributes to improving patient safety and the quality of care [13–15]. Through complaints, patients and relatives mostly want to protect others from experiencing what happened to them [16–22] obtain explanations [19, 21, 22] and apologies [19–22]. Dissatisfaction concerns issues related to the clinical and technical aspects of care [6, 8, 16, 18, 21, 23–25], the relationships among patients, relatives and HCPs (healthcare professionals) [6, 8, 16, 21, 23–26] and the organization and management aspects [6, 8, 16, 21, 24].

In contrast, unsolicited positive feedback such as compliment letters has not been a matter of special interest until recently [1, 27, 28]. Compliment letters is the term used by Gillespie and Reader to qualify “unsolicited positive feedback on specific encounters that patients and their families (…) send via post or email without expecting a response” [1] [p. 484]. To date, studies on compliment letters have been conducted mainly in palliative [29–32] and intensive care settings [33–36]. In palliative care, Aparicio et al. [29] and Centeno et al. [30] reported that relatives valued the care provided: they conveyed messages of support [29], praised team attitudes and professionalism [30], expressed gratitude for the treatment [30] and emotional support provided for the patient [30], and valued the humane atmosphere and holistic care [30]. In intensive care, former patients wrote to praise the care, the expertise of the team and their relational competences [33]. They also recounted their experiences of being in the unit and described their daily lives after discharge [33, 34]. Thanking the HCP was not only an act of conventional politeness but also a way to experience continuity of care [35].

Compliment letters from hospital users have been regarded in these studies as expressions of satisfaction [1, 37] and gratitude toward HCPs [28, 29]. In this context, gratitude has been evaluated in terms of its benefits for both those who express it and those who receive it. Gratitude interventions have been developed to enhance well-being, notably for patients, by writing gratitude lists, practicing grateful contemplation and expressing gratitude to others [38].

Aparicio et al. noted that expressing gratitude has positive effects on well-being and decreases stress and anxiety among HCPs [39]. Gratitude positively impacts feelings such as pride, satisfaction or well-being, which increases motivation and job satisfaction, decreases burnout, and generates reflection [31, 37, 39, 40]. The authors thus argued that gratitude could be used systematically to enhance positive feelings among HCPs and patients. From this perspective, compliment letters can create a sense of reward, recognition, satisfaction and a “mission accomplished” feeling for HCPs [31]. Therefore, these letters can be used for team building, burnout prevention and staff empowerment [31, 39, 40].

In this study, we examined compliment letters written by parents of infants who needed care at birth that were received by a neonatology service. The aims of the study were twofold. First, to investigate compliment letters’ content regarding parents’ experiences and core aspects of what they lived (manifest level). Second, to explore reasons that motivated parents to provide feedback (latent level). Based on compliment letters’ latent content, we sought to identify parents’ underlying needs.

The importance of this study lied in its unique approach. It explored positive feedback from the viewpoint of the sender, examining the functions that compliment letters may fulfill for them rather than their effects on the recipients. Furthermore, compliment letters were analyzed in terms of the overall experience they conveyed, rather than limiting the scope to satisfaction or specific valued aspects of care. Importantly, no similar study has yet been conducted in the neonatology setting.

## Methods

### Study design

This study was designed as a qualitative descriptive research, based on the analysis of 573 compliment letters collected by the neonatology service of Lausanne University Hospital (CHUV) in Switzerland between 2009 and 2020. The parents’ lived experiences were analyzed using framework analysis, while the functions of unsolicited positive feedback were examined through a custom analysis method.

### Setting

As the referral center for a region with 15,000 births/year, the neonatology service of the CHUV admits approximately 800 infants per year [41]. The service includes two intensive and intermediary care units and one special care unit with a total of 40 beds and an average length of stay of 17 days. Patients are infants born prematurely or at full term with health problems, aged between 0 and 28 days and at risk of developing vital function failure.

The service frequently receives compliment letters from parents and relatives. This material was kept rather informally from 1976 to 2009. Since 2009, it has been archived in ring binders.

### Data collection

The head nurse of the service assisted us in collecting the materials during 2021 and 2022. Compliment letters were photographed before being returned. Their contents were transcribed verbatim into Word files. No personal information about the authors of the letters or the children was collected. If such information appeared in the letters, it was anonymized. A database containing the information shown in Table 1 was established. As the data were unsolicited, they lack uniformity, and some of the information listed may be missing.

**Table 1:** Database of compliment letters

letter ID	year of reception	dates mentioned in the letter	time of writing	format	existence of a personalized message for the team	author of the letter	Word count	form	link with previous letters
1.2017	2017	01.03.17	holiday season, one year after discharge	birth announcement	x	parents	56	handwritten	
2.2017	2017	02.01.17		greeting card	x	parents	65	typed	
3.2017	2017	19.10.16–02.03.17		birth announcement		child		typed	
4.2017	2017	03.12.16–23.02.17		birth announcement	x	parents	50	handwritten	
5.2017	2017	08.01.17	3 months after birth	letter	x	parents	251	typed	5.2016
6.2017	2017	30.12.17		birth announcement					
7.2017	2017	20.12.16		greeting card	x	parents	67	handwritten	
8.2017	2017	06.09.17		birth announcement					
9.2017	2017	22.09.17		birth announcement	x	parents	19	handwritten	
10.2017	2017	30.05.17		birth announcement					
11.2017	2017	20.07.17		birth announcement					
12.2017	2017	17.01.17		birth announcement	x	parents	32	handwritten	
13.2017	2017	29.05.17		birth announcement	x	parents	75	handwritten	
14.2017	2017	28.03.17		birth announcement					
15.2017	2017	21.06.17		birth announcement					
16.2017	2017	08.11.17		birth announcement					

We use the term “compliment letter” to refer to our material, which takes different forms: birth announcements, compliment and thank you cards, letters, Christmas and greeting cards, photographs, and e-mails.

### Material

A total of 1425 compliment letters were collected by the neonatology service between 1976 and 2020. Of these, 877 compliment letters received between 2009 and 2020 were considered in the study because this time range corresponds to the period of systematic archiving. To be included in the analysis, compliment letters had to contain a personalized message for HCPs (*n =* 573 letters). The word count ranged from 1 to 573, with a median word count of 47. Table 2 presents two examples.

**Table 2:** Examples of compliment letters

ID	Content	Facsimile
13.2017	A huge thank you to the whole neonatology team for having taken care of our daughter, born at 28 weeks!! Thanks for your kindness, your support, your smiles and your professionalism! She was able to leave the hospital and come back home on Friday! And thanks to you, she is doing wonderfully well today! THANK YOU SO MUCH! You do an amazing job!	
61.2019	A big thanks to the whole team for the care you gave me during my bronchiolitis.	

### Data analysis

Framework analysis was used to explore the compliment letters’ content (first aim of the study). This method, which has similarities with thematic analysis, is suitable for the analysis of large datasets, facilitates teamwork and is appropriate for studies with an experiential focus [42]. Two of the authors, VC and CB, carried out the analysis and relied on the worked example provided by Parkinson et al. [42] to get used to the application of the framework analysis. Framework analysis is composed of five stages [43]. First, researchers *familiarize* themselves with the data. In this stage, VC and CB read and transcribed compliment letters. Second, they *identify a framework*, which implies that they defined categories for coding. VC and CB read the same 10 letters and coded them separately before discussing the categories together to create the framework. They repeated this task until saturation of new categories was reached. Utterances ending with a period were chosen as analysis units for coding. Third, researchers *index* the dataset, which consists of applying the framework to all the data. The letters were analyzed year by year, starting from 2020 back to 2009. Each category was then analyzed to determine subcategories. The coding categories were discussed and agreed upon in team discussions. When disagreement occurred, utterances were discussed until consensus was reached, which allowed us to provide defined standards for the coding book. Fourth, researchers *create charts* that summarize and organize data to obtain an overview of the range of aspects considered for each coding category. Finally, they *map* the analyzed data to find patterns *and interpret* them in relation to the concepts and research questions. The data were sorted based on the combinations of categories they contained to map them and identify themes for interpretation. MAXQDA software was used to analyze the content of the letters.

With respect to the identification of underlying needs that may lead parents to provide feedback (second aim of the study), a custom analysis method was developed. It relied on both the content of the compliment letters and the results of the framework analysis—specifically, the identified themes, their significance, and their presence or absence in the letters. The approach was interdisciplinary, drawing on the theoretical backgrounds of the research team. This custom method involved an inductive and interpretative analysis, combining perspectives from psychology and socio-anthropology. The analysis was conducted by all the authors (VC, FS, BS, and CB), who inferred the parents’ intentions and feelings related to the underlying needs that may have influenced their writing. Combining psychology and socio-anthropology perspectives was particularly relevant for understanding this phenomenon, which encompasses both socio-interactional and psychic motivations.

### Reflexivity

This study is part of a larger project that focuses on matters of interest, concerns and satisfaction of hospital users. The project team consists of a liaison psychiatrist (FS), two social scientists embedded in the same psychiatric liaison service (CB and VC), and a member of the hospital’s direction and expert in institutional communication and complaint management (BS). The research team met regularly, and each member contributed to the analysis according to his or her background.

## Results

Themes concerning parents’ experiences are presented in the first part of the results, followed by a description of parents’ underlying needs. Italics indicate themes, subthemes and types of needs; excerpts of letters are provided for illustration.

### Core dimensions of the parents’ experiences

The framework analysis allowed for the characterization of parents’ experiences through five core themes: *recall of the care and hospital stay; life after the hospital discharge; parents’ feelings; perceptions of HCPs;* and *parents’ gratitude.* For each theme, subthemes were identified.

#### Recall of the care and hospital stay

A core dimension of parents’ experiences identified in the compliment letters was their recollection of the care and hospital stay, with contextual details provided about what they went through when their children were in the neonatology unit.

Specifically, by mentioning the children’s *dates of birth*, they recalled the beginning of the hospitalization. In a few cases, the *date of death* reminded the tragic outcome. Mentions of dates or the time elapsed were cues to situate the *moment of the beginning of care*, the *length of the hospital stay* and the *moment of hospital discharge.*

*Almost two years ago*,* our son was born prematurely at CHUV’s maternity ward. (…) He would spend more than 3 months in your unit*,* then more than 3 months in the neonatal care unit of another hospital to finally join us. (16.2011)*

Parents also described the context of their experiences by recalling their children’s clinical situations, states of health at birth, and possible *pathologies*. They mentioned *weeks of the mother’s amenorrhea* as well as *height and weight at birth*, both categories hinting at their children’s prematurity.

*Our son was born on May 23*,* 2017*,* at 29 WA. He was 42 cm tall and weighed 1 kg 530 g. (27.2017)*

*Fifteen months ago*,* I stayed for 3 weeks at your place because I had esophageal atresia. (54.2015)*

At times, they framed their experiences as an *adventure*, recounting it as a series of events with highs and lows, and potential plot twists.

*However*,* 1 h 30 min after his birth*,* despair replaced the joy of becoming the parents of this little sweetheart*,* our son*,* after he experienced complications at birth (umbilical cord around the neck and forceps)*,* causing a cerebral hemorrhage leading to cardiac arrest. Once our little love was resuscitated*,* he was transferred to CHUV’s neonatology service in a more than critical condition. For us*,* as young parents*,* the nightmare began. A doctor told us that the prognosis was vital*,* that all his organs had been affected*,* and that none of them were functioning properly. Our son was pale*,* intubated*,* and placed on a drip. We did not understand anything. The pregnancy was perfect*,* he was born at full term*,* at 53 cm and 3.300 kg… How was this possible? What happened? Why? (…) Our son spent 41 days in the neonatal unit. Day after day*,* he fought*,* and little by little*,* all his organs began to function optimally. Of course*,* there were up and downs*,* inexplicably intense emotional waves*,* but he made it. (33.2017)*

#### Life after the hospital discharge

In compliment letters, parents recounted how their children’s health has progressed and how they developed as individuals. They reported on their children’s *fitness* and described their *growth*, mentioning their current weight, height and age or stating that they were growing well. The children’s *nutrition* and relationship with food also played a part in the letters as a sign of good health.

*She is doing well*,* eats a lot*,* weighs 5 kg and is 56 cm tall (7.2013)*.

Parents provided in compliment letters feedback on their children’s *medical evolution* since discharge. The children’s conditions were slightly improved, improving or even fully resolved. They mentioned eventual complications or medical interventions.

*Today*,* he is doing perfectly well*,* and his cardiopathy has been completely corrected. (19.2017)*

Parents also placed emphasis on day-to-day experiences, referring to their children’s *qualities*, the developmental *stages* they reached, their *activities* or their *place in the family.*

*A cheerful*,* mischievous little girl who is already learning to walk! (13.2020)*

*Three days a week*,* I follow life at the day care center like big children*,* and my beloved brother never misses an opportunity to teach me a lot of nonsense. (24.2014)*

#### Parents’ feelings

Parents’ feelings regarding the care provided to their children played a significant role in the compliment letters. They reported having been moved by the actions of the *professionals* and expressed both their *feelings* and admiration for them.

*For us*,* the admiration is even greater because*,* with the hindsight of our experience*,* we realize how special the job you have is. (67.2019)*

They also witnessed the impact of past care *situations* by highlighting the difficulties they encountered.

*We have lived the most painful days of our life (…). Today*,* it is only an unpleasant memory*,* which is replaced by so much happiness and joy (…). (31.2013)*

The parents also disclosed their *feelings* toward their *children*, most often saying that they filled them with happiness.

*She thrives more and more*,* and we are very happy to have a wonderful girl in such good health! (23.2011)*

The parents expressed their feeling of *gratitude*, describing what they owed to the team and offering *compliments* to show their appreciation. On special occasions, such as birthdays or visits to the hospital, parents explained that they were reminded of the HCPs, who remained in their *memory*.

*Keep it up*,* we will never forget you! (76.2019)*

#### Perception of the HCPs

Parents’ experiences also encompassed the HCP’s and their ways of being and doing. The compliment letters highlighted a range of *qualities* demonstrated by healthcare professionals, including optimism, commitment and determination, kindness, professionalism and competence, empathy and humaneness, as well as attentiveness and patience.

*Your optimism*,* determination*,* dedication*,* and kindness are without a doubt the magic formula to help these little human beings overcome the difficulties of their extremely brutal arrival in earth life. (40.2014)*

The parents reported their experiences in relation to the HCP’s *actions*, which they valued. For example, they admired when HCPs saved children’s lives, took care of children and their parents, responded to children’s needs, and communicated, listened to and supported parents.

*All of you were there*,* day and night*,* taking care of him and meeting his needs*,* reassuring us*,* playing down our fears*,* encouraging us or simply listening to our worries as parents. (26.2009)*

#### Parents’ gratitude

Lastly, the compliment letters expressed parents’ gratitude, which formed a central part of their experience. They *thanked* the team members for their *qualities* and *actions*, or sometimes without specifying particular reasons.

*A huge thank you to the whole team of doctors and nurses*,* especially Amanda. (31.2019)*

*Many thanks for your care*,* kindness and support. (62.2009)*

### Underlying needs of parents in writing compliment letters

The custom analysis revealed that writing could allow parents to express their need *to thank the team* in the wake of their experiences or to find *closure* regarding the situation, either completely or partially. Writing a compliment letter may also respond to the parents’ need *to take their place in the care* process and *manage their sense of indebtedness* toward the team.

#### Thanking the team

Almost all the compliment letters contained expressions of gratitude. Expressing gratitude could be seen as an act of politeness or as holding a deeper significance for parents, as if thanking the team could ensure that everything will continue to be fine.

*A big thanks to the whole team for having taken such good care of our two little angels*.

*Let’s hope that their journey continues as well as it started. Thank you again. (18.2010)*

#### Closure

With their compliment letters, parents may have needed to symbolically close their children’s medical history and distance themselves from what they had been through. Since the children had recovered, they were no longer patients requiring care. The parents thus needed to affirm that they could begin to live normally and move beyond a life assisted by medical care.

*Many thanks to the entire neonatology team for the care and attention given to our son during his stay at CHUV. Today*,* he is almost two months old*,* and he is 52 cm tall and weighs 3 kg 940 g. He’s growing well*,* and he’s very alert. Thank you*,* a hundred times*,* for everything. (14.2011)*

#### Partial closure

*Partial closure* seemed to be driven by the need to provide feedback and update the team on the children’s progress. The parents sought to share this information with specialists who are able to appreciate the progress made. From the parents’ perspective, the HCPs were well-placed to assess the children’s development, having known them since birth.

*Thank you for all the care during this intense period. The picture is already a bit old*,* now Julia doesn’t need a tube anymore*,* doesn’t require oxygen and she has started to develop. (16.2019)*

#### Taking its own place in the care

Writing a compliment letter may also serve as a way for parents to recount how their children’s hospitalization affected them. After having been backstage during the hospitalization, recalling and sharing their experiences allowed them to express feelings that they might have kept silent or even might not have been able to recognize in the moment.

*Thanks a lot for your help and for taking care of our son during his stay in the neonatology service. We thank you for your patience and kindness. We are sorry if we caused trouble*,* but for us*,* it was a very difficult situation to have our baby in intensive care with many health issues. In addition*,* it was difficult to wait for four very long months until he could leave the hospital. However*,* we improved our French. Thank you! (18.2012)*

During care, the children were the focus, and the parents’ experiences were placed on the back burner. This category of needs highlighted that those parents suffered and that, at some point, felt the need to express what had affected them.

#### Managing the sense of indebtedness

Parents needed to acknowledge that they owed something to the team. What they owed was so significant that the debt could neither be honored nor erased. Indelible bonds between the team, the children and their families may have resulted from this sense of indebtedness. The parents may have found a way to manage this debt by admitting its existence in a letter.

*Four years after her birth*,* Sophie now understands that without you*,* things could have been much more different. Today*,* more than ever*,* we don’t forget you*,* and we say THANK YOU! (33.2020)*

## Discussion

This study showed that compliment letters written by parents and collected by a neonatology service convey more than thanks or other expressions of gratitude. They support parents’ narratives of their experiences, which often refer to a context (*recall of the care and hospital stay*) with protagonists who have feelings (*parents’ feelings*), observe other protagonists (*perception of the HCPs*) and update HCPs on what happened after their encounter (*life after the hospital stay*). In addition to the need to express gratitude (*thanking the team*), recounting their narratives appears to be a way for parents to take leave of the hospital world (*closure*), to provide feedback on the children’s state of health (*partial closure*), to acknowledge the debt they feel they owe to HCPs (*managing the sense of indebtedness*), and to share what they experienced during their children’s care (*taking its own place in the care*).

These results will be discussed from a socio-anthropological perspective taking into account the psychological impact of children’s illness on their parents. We will focus on the underlying needs and the functions that compliment letters might have for them. Then we will discuss the role of narration and the use of writing to make sense of an intense experience. We finally adopt an organizational systemic stance to critically question how compliment letters are used by health care institutions.

Based on the underlying needs identified by the analysis, we can infer different functions that writing compliment letters may have for parents. A wish for normalization may respond to the need for *closure*. When describing their children as being like any other child, doing and enjoying activities as “normal” children do, parents might indeed aim to normalize the situation of their child even though children may suffer from potential consequences of prematurity or from their condition at birth in the long term. Compliment letters might also support parents in their quest for recovery by updating HCPs on the state of health of their children, as it can be inferred from the need for *partial closure.* Moreover, *partial closure* may indicate parents’ willingness to maintain relationships with HCPs. Likewise, the need *to manage the sense of indebtedness* supports the interpretation that parents may seek to keep bonds with HCPs. Indeed, having their children treated by HCPs could possibly induce a feeling of debt, a debt that cannot be paid back [44]. This could have been considered overwhelming [44, 45], and parents might have wished to transform it into positive debt, a debt that one does not try to repay and that enriches and strengthens their identity [46, 47]. Finally, parents may seek to provide HCPs with a testimony of their own journey within the course of their children’s care (*taking its own place in the care).*

Beyond these functions, we can assume that the process of writing compliment letters might be related to magical thinking. Indeed, writing could be considered as a ritual to ensure ongoing protection and that everything will be fine in the future [48, 49]. In our case, parents can read a selection of compliment letters displayed in the neonatology service to support families. Seeing compliment letters recounting positive outcomes for other children might also reinforce the belief in a positive effect of writing a compliment letter.

The experience of neonatal care can sometimes have a traumatic dimension. As some parents wrote, having a child in a neonatology service was like “a storm in a blue sky.” A moment that should have been considered pure joy suddenly turned into anxiety and uncertainty. Writing compliment letters may thus be a means for parents to create their own narrative, adjusting its content, and settling its chronology. According to Frank, who has extensively studied illness experience, illness provokes an interruption in the course of life. Telling one’s story is then a way to find meaning and transform the story into an experience [50]. In a similar vein, for Bendrihen, who works with cancer patients, writing allows patients to restore continuity in a story that was challenged by difficult events or trauma [51]. Narratives, by their capacity to integrate at the same time rupture and continuity, are a way to address the change and permanence of identity that are induced by illness, or, as in our study, the complications related to the birth of the child [52]. Frank uses the term “illness narratives” to describe stories supporting ill people’s meaning making [50]. Compliment letters meet Frank’s definition because they allow parents to make sense of their experience, to reconstruct and tell their story. As highlighted by Frank, illness narratives are addressed to someone. For parents, the HCPs might represent an audience able to understand what they went through. Frank also pointed out that illness narratives circulate and influence ways of telling. As previously mentioned, a selection of compliment letters is displayed by the HCPs on the walls of the neonatology service considered in our study. It is probable that being surrounded by this material during their children’s hospital stay may have prompted parents to send one. It is also likely that parents wish to share their stories to support other parents. Moreover, the contents of compliment letters might also be influenced by what parents read in the letters from parents who expressed their satisfaction before them (displayed material). In this regard, the unsolicited dimension of compliment letters, which is a particularity highlighted in the literature, can be questioned in certain settings such as that of our study.

Lastly, Frank distinguished types of illness narratives based on their plot. He pointed out restitution narratives as the preferred type of illness narratives because they tell stories in which ill people have recovered. On the opposite are chaos narratives, stories without narrative order describing a life that is never getting better and quest narratives, stories in which something can be gained from the experience of illness [50]. With regards to compliment letters, the experiences recounted by parents and the underlying needs identified present characteristics of restitution narratives. When parents describe their children’s experiences and state of health during the hospital stay (*recall of care and hospital stay*), how it impacted them (*parents’ feelings*), and how it contrasted with their daily life after the hospital stay (*life after the hospital discharge*), they recount a story of recovery.

From a hospital systems perspective, the parents’ needs and perhaps desire to share their experiences may also echo the expectations of HCPs and institutions. HCPs indeed expect to be recognized for their work [53], and institutions want to ensure and enhance their engagement and motivation. Current trends in work and human resources management advocate for employees’ well-being to enhance engagement and productivity and reduce stress and burnout [54–56]. According to positive psychology, as gratitude contributes to well-being [57], transmitting compliment letters to HCPs is one of the ways to achieve these goals [30, 39, 58, 59]. This reading raises several issues. First, if HCPs benefit from gratitude, it should not only be up to patients and relatives to fulfill this task [53]. HCPs need genuine recognition from their peers, superiors, and institution, not only by communicating general messages of recognition for their work, but also by taking initiatives to improve their work conditions. Indeed, as stressed by Wood and Skeggs, there is a certain contradiction in promoting staff recognition and well-being and, at the same time, implementing austerity policies and putting more pressure on them [60]. HCPs also need good working conditions, the possibility of “speak up” on every matter, adequate human and material resources, predictable schedules and limited overtime [61–63]. In this sense, patients and relatives’ compliment letters are not enough as a means of gratification for HCPs.

Lastly, the informational value of the experiential content of complaints is now widely recognized as a full-fledged factor contributing to health care quality [64]. Negative experiences receive institutional and scientific attention, whereas positive experiences are less studied and understood as a source of information conveying not only gratitude but also matters of concern and challenges faced by hospital users. Positive feedback contains information that can be useful for hospital stakeholders. Considering them only as signs of gratitude and a means to increase staff members’ well-being and galvanize them has the effect of ignoring the experiential dimension they contain.

### Strengths and limitations of the study

This study adopted an original approach by analyzing both the manifest and latent content of compliment letters. By highlighting the experiences described in these letters and identifying the underlying needs that motivated their writing, the study broadens the understanding of compliment letters beyond their presumed function of expressing gratitude. It also emphasizes the potential significance these letters may hold for their authors. This perspective opens new avenues for exploring the dynamics behind compliment letters and provides valuable insights for health services and hospital management in interpreting and responding to this type of feedback.

However, as the study relied on unsolicited material, the form and content of the compliment letters varied considerably. As a result, the data was relatively heterogeneous, which limited the scope of the observations, particularly concerning the extent to which parents’ experiences were reflected in the letters. Moreover, since the analysis of underlying needs was based on latent content, parents’ varying levels of comfort with writing likely influenced the possibility to identify deeper motivations.

## Conclusion

This investigation based on an interdisciplinary approach of compliment letters addressed to a neonatology service showed that when parents wrote such letters, they mostly expressed how they experienced the hospitalization of their children, how it impacted them and how they managed to make sense out of this experience. Thus, it is likely that parents wrote letters for HCPs as well as for themselves. Compliment letters have other functions in addition to expressing gratitude. They allow parents to sustain relationships with HCPs, support the normalization of their children’s situation and their recovery or provide testimony of experiences. Focusing only on the dimension of gratitude entails the risk of altering the meaning of compliment letters and missing parts of what parents wish to share and communicate. It is important to acknowledge that compliment letters contain hospital users’ experiences of care in addition to expressions of gratitude and positive evaluations of HCPs. Compliment letters should be recognized as part of a reconstructive narrative and sense-making process for those who write them, conveying important information not only about the writers themselves but also about healthcare professionals, the relationships formed during the hospital stay, hospital operations, and the broader healthcare organization.

As hospital users’ experiences are thought to contribute to health care quality, it matters that health services consider the information that compliment letters convey.

Future studies could further explore the functions of compliment letters by conducting interviews with patients or relatives who provide such unsolicited feedback. Additionally, the significance and meaning attributed to positive feedback by healthcare professionals should be examined to understand how it is received and interpreted within clinical settings.

## Data Availability

The dataset analyzed during the current study is not publicly available due to confidentiality issues and the fact that the analyzed compliment letters are the property of Lausanne University Hospital’s neonatology service.
